# Frequently Asked Questions in Patients With Adrenal Insufficiency in the Time of COVID-19

**DOI:** 10.3389/fendo.2021.805647

**Published:** 2021-12-24

**Authors:** Chiara Sabbadin, Corrado Betterle, Carla Scaroni, Filippo Ceccato

**Affiliations:** ^1^ Endocrine Disease Unit, University-Hospital of Padova, Padova, Italy; ^2^ Department of Medicine (DIMED), University of Padova, Padova, Italy; ^3^ Department of Neuroscience (DNS), University of Padova, Padova, Italy

**Keywords:** COVID-19, glucocorticoid treatment, immune response, adrenal insufficiency, Addison disease

## Abstract

Adrenal insufficiency (AI) is a life-threatening disorder, with increased morbidity and mortality, especially in case of an acute illness that can increase the requirement of cortisol. A novel infectious disease, termed Coronavirus Disease 2019 (COVID-19), appeared in 2020. Therefore, AI patients are experiencing a novel challenge: the risk of infection. In our experience, a prompt contact to the Endocrine center (with a telemedicine consultation) and a full awareness of diseases (cortisol deficiency, COVID-19 and the self-management of an adrenal crisis) are important to motivate patients. Vaccine is an effective treatment to prevent hospitalization and aggressive course of COVID-19. Some patients manifest challenges due to inequitable access and vaccine hesitancy, resulting in a delay in the acceptance of vaccines despite the availability of vaccination services. Therefore, an effort of all physicians must be conducted in order to advise patients with AI. In this short review, we try to answer some frequently asked questions regarding the management of patients with AI.

## Introduction

In early 2020, the world experienced the global pandemic of the Severe Acute Respiratory Syndrome (SARS) Coronavirus 2 (SARS-CoV-2) (1). From a clinical point-of-view, the Coronavirus Disease 2019 (COVID-19) ranges from asymptomatic cases, to patients with mild/self-limiting respiratory tract illness, up to subjects with severe progressive disease with pneumonia and multi-organ failure (2, 3). Containment measurements were progressively expanded, combined with the use of personal protective equipment, enforcing social distancing, isolation and quarantine of all positive cases and their relatives. Nowadays, several vaccines (including those with novel mRNA technology) are validated by national and international drug regulation agencies (4), and are effective in the control of the progression from mild symptoms to severe disease (5).

Adrenal insufficiency (AI) is defined as an insufficient production/secretion of glucocorticoids (GC) and/or mineralocorticoids (6), especially in primary AI (6). On the contrary, central AI is characterized by inappropriate ACTH secretion (7). People with AI are facing their primary disease and the risk/fear of COVID-19 infection (8). Therefore, a complete awareness of diseases (AI and COVID-19) and motivation about self-management are of paramount importance in patients.

In this short review, we propose an updated state-of-art regarding the management of patients with AI.

## Is Mortality and Infectious Risk Increased in Patients With AI?

Conflicting data exist about the rate and the causes of mortality of patients with Addison Diseases (AD, the most common autoimmune AI) (9–12).

The first study, based on data from the National Swedish Hospital Register, reported that the risk for mortality in patients with AD was 2.19 in men and 2.86 in women, mainly due to cardiovascular diseases, cancers, infectious diseases and diabetes mellitus (DM) (9). An inappropriate GC replacement (both excess or inadequate increment of doses in response to stress conditions) may be responsible for the increased mortality. These results were confirmed in AD patients admitted to hospitals: mortality rate was 2.9 for women and 2.5 for men, up to 4.6 in autoimmune polyglandular syndrome type 1 (APS-1, a rare AD characterized by hypoparathyroidism and chronic mucocutaneous candidiasis, due to mutations in the autoimmune regulatory gene) (10). A subsequent Norway study did not confirm these data, except in patients with young onset of AD, probably affected by APS-1 (11). A critical evaluation of these studies revealed some bias, as the use of general mortality registers in which it was not possible to confirm the accuracy of the diagnosis of AD (12).

A study in 2017 reported that patients with AD had a reduced natural-killer cell cytotoxicity that impairs the early recognition of infected cells in the respiratory tract (13). This impairment in anti-viral immune defense may contribute to the increased rate of infections (not only SARS-CoV-2). However, a UK study with 1580 patients with AI (AD or congenital adrenal hyperplasia, CAH) showed that patients with GC treatment had an increased risk of respiratory, urinary or gastrointestinal infections and of prescription of antimicrobials respect to CAH without GC therapy (14). A recent paper demonstrated that patients on conventional GC therapy had a pro-inflammatory state and a weakened immune defense; a normalization of the immune cell profile and a reduction of infections was observed after the restoration of the physiological circadian cortisol rhythm with modified-release hydrocortisone (HC) (15). A Swedish cohort reported an increased relative risk of death (respectively 28% and 10%) in 226 patients with diabetes (Type 1 and 2) and AD matched with 1129 controls with only diabetes, especially due to diabetic complications and infectious diseases (16). The authors suggested that adrenal crisis could be a contributing factor to this increased mortality.

## Is COVID-19 Risk and Management in Patients With AI Different From General Population?

Patients with AI are considered “clinically vulnerable” for their increased risk of infections, that could lead to poor prognosis and death due to adrenal crisis (14). A recent Expert Opinion of the Italian Society of Endocrinology (SIE) suggested that not only the aetiology of the AI, the length of follow-up, the patient’s age or the expected adherence to therapy, but also the comorbidities must be carefully evaluated during substitutive treatments (17), because mimicking cortisol rhythm can reduce recurrent infections (15). In the last months, a task force of the SIE (8) and the European Society of Endocrinology (ESE) (18) published some recommendations regarding GC replacement in patients with AD infected with SARS-COV-2 according to the stage of the disease. Others proposed strategies to improve patients’ education to manage high-risk situations, to prevent adrenal crisis (19).

During the first COVID-19 wave, two cross-sectional studies reported a low prevalence of infection among AD patients, and COVID-19 disease severity similar to healthy controls in 393 patients with primary and secondary AI referring to Italian centers. An important emotional impact was found in some patients requiring an up-titration of the usual GC replacement: patients’ education about infection-related risks and adequately self-adjustment of replacement therapy were fundamental to prevent acute events and complications (20, 21).

Another study reported that among 159 patients taking steroid replacement therapy for pituitary disease, 30 patients (18.9%) reported symptoms of COVID-19 infection, but only two of the seven patients tested for COVID-19 infection resulted to be positive (22). Finally, a recent longitudinal survey study performed in 2 tertiary medical centers of the US confirmed a lower prevalence of COVID-19 infection in AI patients compared with overall prevalence (1.8% versus 7.9%, respectively) (23). All infected patients reported mild symptoms and were managed at home.

Based on the available data, there is no evidence that patients with both primary and secondary AI have an increased risk of infection and disease. However, these conclusions could not be extended to patients with APS-1: their primary immunodeficiency lead to the development of young-onset multiple autoimmune disorders (24). APS-1 is associated with an increased mortality from infections and from cancers in comparison to the general population (24, 25). Considering the low mean prevalence of APS-1 (about 10 cases per million inhabitants) (26), only 24 cases of APS-1 patients from seven countries who were infected with SARS-CoV2 has been reported until now (27, 28). 20 patients were hospitalized, 15 showed severe complications requiring admission to an intensive care unit (ICU) and 4 of them died. Interestingly, beyond the underlying peculiar condition (genetic, age and AI), in APS-1 patients the pre-existence of autoantibodies (auto-Abs) neutralizing most type 1 interferons (IFNs), key immune regulators against viral infections, confer a very high risk of developing critical COVID-19. A recent study reported these auto-Abs in about 10% of cases of severe pneumonia in the general population (27). Infected patients with APS-1 should be always hospitalized promptly to evaluate the best management according to the severity of the disease and to their pre-existing risk factors.

**Figure 1:**
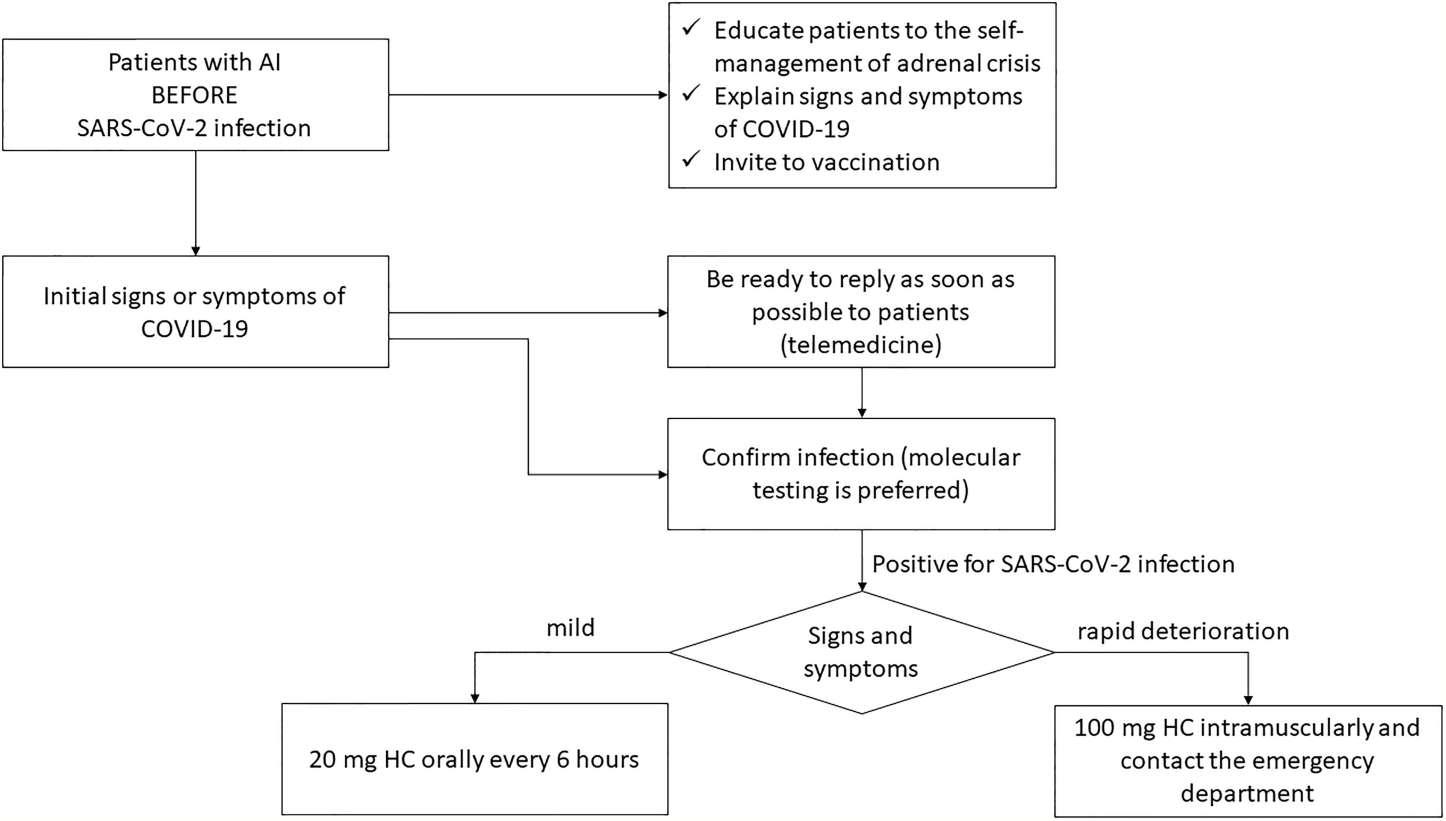
Clinical management of patients with AI and SARS-CoV-2. SARS-CoV-2, Severe Acute Respiratory Syndrome Coronavirus 2; COVID-19. Coronavirus Disease 2019; HC, hydrocortisone.

In case of Sars-CoV-2 infection, the management of patients with AI should follow the sick-days rules, as reassumed in **Figure 1**. In case of signs or symptoms of infection, all patients with AI are encouraged to prompt contact their referral Endocrinologist in order to adjust GC dose and to being advise in case of hospital admission (29). The clearance of HC significantly drops during critical illness: the SIE/ESE recommendations are to administer 20 mg HC orally every 6 hours in case of asymptomatic disease or only uncomplicated mild symptomatic infection (sore throat, mild cough, without headache, vomit, diarrhoea, or fever <38°C) (8, 18). Then, in case of clinical deterioration (incoming hypotension, persistent cough, increased respiratory rate > 30 breaths/minute or SpO2 <93%), the self-administration of 100 mg HC and the contact with the emergency department are suggested (8, 18). In children, 2 mg/kg or 50 mg/m^2^ of HC every 6–8 h intramuscularly, subcutaneous or intravenous, combined with the correction of hypovolemia (0.9% up to 60 mL/kg within 1 hour) and hypoglycaemia, are suggested (6, 30, 31). Beyond the adjustment of GC therapy during COVID-19, patients with AI should reduce their risk to get the SARS-Cov-2 infection through social distancing, the use of masks, hands cleaning with dedicate gel, the choice of work-from-home if possible (18, 32).

## How Should We Diagnose AI at the Time of COVID-19?

Several drugs and conditions can affect hypothalamic-pituitary-adrenal (HPA) axis and induce primary or secondary AI, during or after SARS-Cov-2 infection. In a real-life clinical setting, physicians tend to use GC in most critically ill patients, especially in those affected by acute respiratory distress syndrome (ARDS). Daily GC doses in patients with community-acquired pneumonia in ICU are 32–40 mg of methylprednisolone equivalent daily in the majority of randomized controlled trials reported in a Cochrane review (33). At these doses, GC inhibit immune responses and pathogen clearance, but also suppress lung inflammation. GC administration, titration, duration, or underlying disease are not able to predict AI after GC withdrawal (34). The rationale of GC use in COVID-19 infection is to reduce the abnormal immune reactivity that induce lung damage and progression to ARDS more than uncontrolled viral replication (35). In July 2020 the RECOVERY trial reported that 6 mg of dexamethasone (the most potent synthetic GC) for 10 days is effective in reducing 28-day mortality among patients who were receiving mechanical ventilation or oxygen alone (36).

In addition, the co-administration of antiretroviral drugs may trigger drug-interactions and enhance the exposure to GC, metabolized through the CYP3A pathway (37), the dominant isoenzyme of the hepatic cytochrome P450 system and the primary metabolic step for the degradation of GC (37). Several reports in HIV-infected patients have documented an impairment of HPA axis in patients treated with GC and ritonavir (38, 39): it reduce the activity of CYP3A4 enzymes, increasing GC levels. Most of ritonavir-associated AI have involved fluticasone, an inhaled GC (40–42). IFN-α is used to treat chronic viral infections: it suppresses CYP3A4 expression in human hepatoma cells (43) and alter the expression of constitutive and inducible CYP3A genes in well-differentiated male rat hepatocytes in culture (44). In humans, a flat diurnal ACTH curve and cortisol slope has been observed after IFN-α/ribavirin administration (45). Finally, IFN-β has been shown to modulate the induction of cytochrome P450 enzyme in mice (46).

Viral infection can induce AI directly. During previous SARS outbreak in 2002-2003, up to 40% of the patients showed low basal and post-synacthen cortisol levels, suggesting a direct negative effect on corticotroph cells (47). A primary adrenal injury consistent with bilateral adrenal haemorrhage has been reported in several patients with Covid-19 infection, especially in those with positive antiphospholipid antibodies (APA) (48, 49). In an autopsy study that described 28 different patients, half adrenals presented microscopic alterations: 7 necrosis (generally ischemic), 4 cortical lipid degeneration, 2 hemorrhage and one unspecific focal adrenalitis (50). Despite exogenous GC, critically ill patients may present a relative AI termed Critical-illness Related Corticosteroid Insufficiency (CIRCI), secondary to higher levels of IL-6, IL-10 and TNF-α (51). CIRCI not indicates strictly a pituitary or adrenal injury, but rather a condition of relative cortisol insufficiency resulting from inadequate GC-mediated anti-inflammatory response (52).

Diagnosis of AI is based upon low morning cortisol levels and, in selected cases, to dynamic tests (6, 53). In case of a pandemic outbreak, out-patient visits or blood collection could be a source of viral spreading: during Sars-CoV-2 waves an effort to limit face-to-face consultations has been proposed (32). Salivary sample is a stress-free tool to measure cortisol, suitable for out-patients who can mail it to the referral center (54). A paper of the ESE reported some concerns regarding salivary cortisol, due to the potential contamination of laboratory staff (55), however no study are reported in this situation. Even if it has been never used, also dynamic tests can be performed without an Endocrine clinic, using salivary cortisol (56) and intramuscular ACTH administration (57).

## Can COVID-19 be a Trigger for Autoimmune Diseases?

Autoimmune diseases are multifactorial: the concomitant presence of genetic, epigenetic, exogenous and endogenous factors is required for their development (58).

The role of genetic factors derives from the observation that autoimmune diseases are more common in peculiar ethnic groups or in families: the genes involved are mainly those related to the major histocompatibility complex. However, the genetic predisposition is in general a condition “*sine qua non*” and a) the discordance in identical twins; b) the appearance of the disease in a minimal part of the “genetically susceptible” subjects; c) the diversity of frequency in individuals of the same race living in different geographical areas, argues in favour of the existence of other factors. The main endogenous factors are gender and age. It is a common observation that females have a greater predisposition to autoimmune diseases than males (from 2:1 up to 10:1), and it seems to depend both by the direct action of sex chromosome genes, and by the concomitant hormonal status. Regarding age, some autoimmune diseases favour adults (Hashimoto thyroiditis, systemic lupus erythematosus, Sjogren’s syndrome, AD), the elderly population (pernicious anemia, polyarteritis nodosa) and some present a paediatric onset (type 1 DM, celiac disease, Kawasaki’s disease [KD], type 1 autoimmune hepatitis, APS-1) (59).

The concept of “exogenous factor” can be attributed to infections, chemicals, iodine, radiations, drugs, foods, trauma, additives, smoke, pollution and socio-economic situations. There are many indirect data that support a relationship between viruses and autoimmune diseases; however, the direct data are limited to HBV and panarteritis nodosa; HCV and cryoglobulinemia; rotavirus infection and celiac disease, enteric viruses and type 1 DM, herpesviruses and systemic lupus erythematosus, rheumatoid arthritis or adult-onset Still’s disease (59).

COVID-19 is a new condition and little is known about the immunological changes that occur in the infected individuals. Viral infections stimulate a vigorous immune response, with a cascade of events involving both the innate and adaptive immunity. In addition, viruses can break immunological tolerance and induce autoimmunity by bystander activation, epitope spreading or molecular mimicry. The last occurs when similarities between foreign- and self-peptides favour an activation of autoreactive T or B cells by foreign derived peptides in a genetic susceptible individual. Several studies have documented very high plasma levels of cytokines and chemokines during Sars-Cov-2 infection. IL-1β and TNFα promote Th-1 and Th-17 responses, contributing to high levels of pro-inflammatory cytokines in the context of a cytokine storm syndrome (59). SARS-CoV-2 shares some sequences (GSQASS, LNEVAK, and SAAEAS) with three proteins present in the brainstem respiratory pacemaker: it might account for an autoimmune disease with depression of respiratory pacemaker and it may induce an autoimmune pulmonary damage (59). Several studies demonstrated immunological (as spike protein) and clinical similarities between COVID-19 and hyperinflammatory diseases, leading to the hypothesis that SARS-CoV-2 infection might trigger autoimmune responses in genetically predisposed subjects (59, 60). In the first period of SARS-CoV-2 infection various autoimmune manifestations, including neurologic demyelinating syndromes, autoimmune cytopenias and thrombotic events, were reported (59, 61). APA and APA-related syndrome associated with SARS-CoV-2 infection was evaluated on overall 4273 patients and was found to be present in 515 cases, especially in ICU. On the other hands, most individuals with APA do not experience thrombotic events (62). Immune thrombocytopenia associated with SARS-CoV-2 infection was described in about 30% of the infected patients (62). Acute inflammatory neuropathies resembling Guillain-Barrè syndrome have been reported in 48 patients with COVID-19, a Miller-Fisher syndrome was developed in 4, few cases developed an acute disseminated encephalomyelitis or myelitis (62). Autoimmune hemolytic anemia (AIHA) was described in 14 patients with COVID-19: AIHA could be induced by a molecular mimicry between the viral spike protein and ankyrin-1, a membrane protein of erythrocytes (62). Systemic lupus erythematosus associated with SARS-CoV-2 infection was very rare and described in 6 case reports. A vasculitis associated to anti-neutrophil cytoplasmic antibodies related to SARS-CoV-2 infection is reported in 3 cases (62). Skin lesions reported in COVID-19 patients were classified into 4 groups: exanthema, vascular, urticarial and acro-papular eruptions (62, 63).

The paediatric population appears to be less affected than adults that develop severe SARS-CoV-2 infection. This can be due both to the decreased level of maturity and function of ACE2, and differences in the immune response. Nonetheless, since 2020, paediatricians began reporting cases of children with fever and signs of systemic inflammation with features in common with KD. Compared with the classical KD, newly diagnosed KD-like patients were older and had more signs of cardiac involvement, shock and required more frequently higher steroid treatment. These patients manifested also gastrointestinal symptoms, which are uncommon in typical KD, and very high levels of procalcitonin. KD-like syndrome was confirmed in 1888 patients with an age from 4 months to 35 years (62).

## Is There an Increased Risk to Develop Other Autoimmune Diseases After COVID-19 Infection or Vaccination in Patients With AI? A Personal Experience

Patients with isolated autoimmune AD or with polyglandular diseases ask if they are at increased risk of developing new autoimmune disease after COVID-19 infection or after vaccination against COV-19. Patients affected by one or more autoimmune diseases are at risk to develop other autoimmune diseases (58). In addition, it is important to remember that COVID-19 infection can induce autoimmune diseases in the general population. Nevertheless, in patients with AI followed at the Endocrinology of Padova (202 primary AI and 134 central AI) we did not document new-onset autoimmune diseases during or after COVID-19 infection or 6-months after vaccination (we use only mRNA-based vaccine according to the recommendation of the Italian Institute of Health). Furthermore, to our knowledge, there are not so far published cases describing the development of new autoimmune disorders in patients with autoimmune AD after COVID-19 infection or vaccinations. Obviously, the post-vaccination observation period is limited to 6 months. Vaccinations started in Italy at the end of December 2020 (starting with health employers), in February 2021 it was proposed to frail patients (as AI). At the best of our knowledge, one case of vaccine-induced primary AI has been reported after a bilateral adrenal haemorrhagic infarction due to bilateral vein thrombosis in a patient with vaccine-induced immune thrombotic thrombocytopenia (64).

Regarding the management of substitutive treatment in patients with AI who will receive a COVID-19 vaccine, a recent survey of the Pituitary Society reported that 36% of physicians recommend an increase in GC dosage with the first injection; the others plan to increase replacement therapy in case of fever or vaccination-related symptoms (65).

## Conclusions and Future Perspectives

Patients with AI could present an increased risk of COVID-19; however, the severity of the disease is mainly due to an inappropriate and prompt GC treatment rather than an increased infection susceptibility (which can be real only for the rare patients with APS-1).

Given the current state of the art, we think that vaccine is a safe procedure, and the patients with AI that hesitate to receive the COVID-19 vaccination should be carefully advised that viral infection or the vaccine can produce autoimmune diseases in rare cases, and on the contrary the vaccination is protective against a disease with a high-risk of hospitalization and mortality, especially in frail patients as those with AI.

## Author Contributions

FC, CSa, and CB: Literature review- original draft, review and editing. CB and CSc: Supervision, writing - review & editing. All authors contributed to the article and approved the submitted version.

## Funding

Project Cariparo COVIDIMED 2020.

## Conflict of Interest

The authors declare that the research was conducted in the absence of any commercial or financial relationships that could be construed as a potential conflict of interest.

## Publisher’s Note

All claims expressed in this article are solely those of the authors and do not necessarily represent those of their affiliated organizations, or those of the publisher, the editors and the reviewers. Any product that may be evaluated in this article, or claim that may be made by its manufacturer, is not guaranteed or endorsed by the publisher.
